# Anesthetic Management and Considerations of Diaphragmatic Pacemaker Placement for Cervical Spine Injury: A Case Report

**DOI:** 10.7759/cureus.95441

**Published:** 2025-10-26

**Authors:** Sarah Sun, Dylan Irvine, Gordon Hubbell, Raul Bermudez-Velez, Imani Thornton

**Affiliations:** 1 Anesthesiology, HCA Florida Westside Hospital, Plantation, USA; 2 Anesthesiology, HCA Florida Kendall Hospital, Kendall, USA; 3 Anesthesiology and Critical Care, HCA Florida Westside Hospital, Plantation, USA

**Keywords:** diaphragmatic pacing, diaphragmatic paralysis, high cervical spinal cord injuries, perioperative anesthesia service, perioperative patient safety

## Abstract

High cervical spinal cord injuries (SCIs) can lead to diaphragmatic paralysis, necessitating long-term mechanical ventilation. Diaphragmatic pacemakers (DPs) offer an alternative by stimulating the phrenic nerve to restore diaphragmatic function, thereby improving quality of life and reducing ventilator dependence. However, anesthetic management for DP placement presents unique challenges due to the need to preserve diaphragmatic activity and manage autonomic instability. This case reports a 35-year-old male with complete C4 SCI resulting in quadriplegia and respiratory failure requiring mechanical ventilation. After multiple interventions, including spinal fusion and tracheostomy, he underwent DP placement to reduce ventilator dependency. The patient was successfully transitioned to DP postoperatively and discharged from the hospital on room air via a tracheostomy collar. DP is a valuable intervention for high cervical SCI patients but requires anesthetic strategies that preserve diaphragmatic function and manage common complications such as pneumothorax, autonomic dysreflexia, and ventilator challenges with abdominal insufflation. Avoiding muscle relaxants, continuous diaphragmatic monitoring, and proactive hemodynamic management are essential. The literature highlights rare but serious device-related complications, emphasizing the importance of vigilant intraoperative monitoring and interdisciplinary planning. As DP becomes more accessible, particularly in community hospitals, anesthesiologists must be prepared to navigate its perioperative challenges. This case underscores the importance of tailored anesthetic approaches to support safe and effective DP implantation.

## Introduction

Diaphragmatic paralysis is a feared complication of a high spinal cord injury (SCI). The diaphragm is innervated by cervical nerves C3 to C5. Injuries to this area or higher may cause diaphragm weakness or paralysis, thereby affecting respiratory function. There are approximately 11,000 cases of SCI annually. Half of these patients develop tetraplegia, with 4% requiring long-term mechanical ventilation [1].

Standard therapies following acute high cervical injury are tracheostomy and ventilation. Diaphragmatic pacing stimulates the phrenic nerve to cause diaphragm contraction, which helps to generate breaths, providing minute ventilation. The placement of a diaphragmatic pacemaker (DP) can facilitate ventilator weaning and eventually aid in patients' liberation from mechanical ventilation. This improves the quality of life for affected patients, decreases the risk of respiratory infection, reduces the need for external power sources, and increases the ease of mobility and transportation [2,3]. Placement of the DP poses many challenges for anesthesia. Understanding the placement, challenges one may face, and how to manage these patients perioperatively are critical in optimizing patient outcomes.

We present a case of a patient with a high SCI with a DP placed in our community hospital. This case report is an educational opportunity for other facilities as they navigate anesthetic management for patients undergoing a DP placement.

## Case presentation

**Figure 1:**
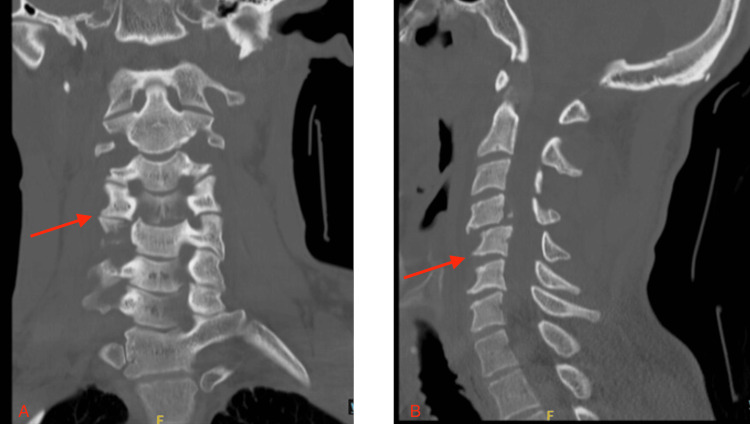
Cervical spine computed tomography (CT) anterior-posterior (A) and lateral (B) views. C4 inferior articular process with displacement of fracture fragment, C4 posterior inferior endplate with traumatic 5 mm anterolisthesis of C4 and C5. Trace anterior wedge deformity of the C5 vertebral body with acute fracture deformity.

We present the case of a 35-year-old male who was found unresponsive with traumatic injury from a suspected fall. He was brought to the emergency department as a Level 1 trauma patient. The primary survey revealed a Glasgow Coma Scale (GCS) score of 15 with a patent airway, and an extended Focused Assessment with Sonography for Trauma (eFAST) was negative for free fluid. He was unable to move his upper and lower extremities and lacked sensation below the neck. Imaging and clinical assessment confirmed a complete C4 spinal cord syndrome with a Grade 5 spinal cord contusion and cervical spine fracture, resulting in quadriplegia (Figure 1).

His hospital course was complicated by multiple sequelae of high cervical SCI, including acute respiratory failure requiring mechanical ventilation, initial neurogenic shock requiring vasopressor support for two days, bradycardia, neurogenic bladder, aspiration pneumonia, and lactic acidosis secondary to poor perfusion from neurogenic shock. He underwent multiple surgical and supportive procedures during his care, including anterior cervical corpectomy, posterior C3-C7 instrumented fusion, partial C3-C6 bilateral decompressive laminectomies, bronchoscopy, tracheostomy placement, and percutaneous endoscopic gastrostomy (PEG) tube placement. The patient was weaned off pressure support and was subsequently scheduled for laparoscopic DP placement as a long-term strategy to reduce ventilator dependence and improve respiratory autonomy.

On the day of surgery, the patient was hemodynamically stable, off vasopressor support, and not receiving any intravenous infusions. A radial arterial line was placed in the intensive care unit (ICU) for continuous hemodynamic monitoring. Preoperatively, the patient received 4 mg of midazolam. He was then transported from the ICU to the operating room (OR) with standard American Society of Anesthesiologists (ASA) monitors in place. Ventilation was maintained via manual bag-mask ventilation through his tracheostomy.

In the OR, the patient was positioned supine and monitored with standard ASA monitors, including electrocardiogram (ECG), pulse oximetry, capnography, temperature, and invasive arterial blood pressure via the radial line. General anesthesia was induced with intravenous fentanyl and propofol. Neuromuscular blocking agents were deliberately avoided to preserve diaphragmatic function for intraoperative testing. Anesthesia was maintained with sevoflurane at an end-tidal concentration of 1.2%. Bispectral Index (BIS) monitoring was used throughout the case, with values maintained between 40 and 60.

Intraoperative hemodynamic support included administration of 300 micrograms (mcg) of phenylephrine, 20 milligrams (mg) of ephedrine, and 1 gram (g) of calcium chloride. Mechanical ventilation was delivered using synchronized intermittent mandatory ventilation (SIMV) with a tidal volume of 500 mL and a respiratory rate of 18 breaths per minute. End-tidal carbon dioxide (EtCO₂) levels were maintained between 38 and 40 millimeters of mercury (mmHg).

To facilitate diaphragmatic mapping, the patient was intermittently disconnected from the ventilator. Two subcostal trocars were placed bilaterally to allow direct stimulation of the diaphragms. Stimulation was applied to identify optimal electrode placement sites based on stimulation parameters with a neurostimulator representative. After confirming appropriate diaphragmatic responses without cardiac interference, four electrodes were successfully implanted: two on each hemidiaphragm. All electrodes were tested intraoperatively and demonstrated effective stimulation with robust diaphragmatic contraction. No intraoperative complications were observed aside from transient hypotension. The total surgical duration was 3 hours and 15 minutes.

Postoperatively, the patient was transferred back to the ICU in stable condition. Pain was well controlled with acetaminophen alone, and no opioids were required. His postoperative course was uneventful. He underwent a structured diaphragm pacing adaptation protocol under the supervision of the surgical team and the neurostimulator programmer. Pacing parameters, including amplitude, pulse width, frequency, and duration, were systematically adjusted based on the patient's diaphragmatic response. These parameters were gradually increased over time, with continuous monitoring of respiratory function, gas exchange, and overall comfort. The patient demonstrated excellent adaptation to diaphragm pacing without evidence of respiratory distress, hypercapnia, or diaphragmatic fatigue. He was discharged on postoperative day 23, breathing spontaneously on room air through a tracheostomy collar that provided diaphragmatic support, with no further need for mechanical ventilation.

## Discussion

Long-term mechanical ventilation carries significant risks for patients who have suffered high SCI. Respiratory complications such as pneumonia, atelectasis, and respiratory failure are common and represent the leading causes of mortality [4,5]. One study found that ventilator dependence was the strongest predictor of reduced one-year survival post-discharge [6]. DP presents a promising alternative to prolonged mechanical ventilation in patients with high cervical SCI. While DP is associated with improved quality of life and reduced ventilator dependence, its placement poses distinct anesthetic challenges that demand meticulous planning and vigilance. Intraoperatively, the anesthesiologist must manage ventilation during laparoscopic surgery without the use of neuromuscular blockade to preserve diaphragmatic function for intraoperative mapping, which can limit the anesthesiologist's ability to optimize surgical conditions and complicate ventilatory management.

In our case, anesthetic management was guided by literature detailing optimal practices for DP implantation. Our anesthetic approach avoided muscle relaxants to preserve diaphragmatic function, used BIS to guide anesthetic depth, and incorporated dynamic ventilator weaning to test pacemaker efficacy. Several reports underscore the importance of avoiding neuromuscular blocking agents during DP placement to preserve diaphragmatic function for intraoperative testing. Our case followed this principle to allow real-time mapping and verification of diaphragm stimulation. This approach is consistent with other providers, who avoided muscle relaxants during laparoscopic DP placement to allow diaphragmatic mapping and reported favorable outcomes with desflurane and remifentanil maintenance [7]. Alegret et al. (2019) reported on 16 patients who underwent DP placement, five of whom had SCI [8]. The most frequent intraoperative complication was hypotension (50%), which aligns with our patient's episode of hypotension managed effectively with phenylephrine and ephedrine. Their findings emphasize the importance of proactive hemodynamic monitoring and management, especially in patients with autonomic dysfunction [8,9].

Patients with high-level SCI are at risk for autonomic dysreflexia (AD), a potentially life-threatening condition that can occur in individuals with SCI, typically at or above the T6 level. It is characterized by an exaggerated sympathetic response to noxious stimuli below the level of injury. This dysregulated sympathetic response can manifest intraoperatively as sudden, severe hypertension and bradycardia, necessitating prompt recognition and management [10]. Our patient, who sustained a high cervical SCI, was at theoretical risk for developing AD during and after DP placement. Although he did not exhibit signs of AD perioperatively or postoperatively, this potential complication remained an important consideration in his anesthetic and surgical management. AD typically emerges after the resolution of spinal shock and the return of spinal reflexes, often within weeks to months following injury [9]. Since our patient had progressed beyond the acute phase and had regained some reflex activity, careful monitoring was warranted. Intraoperative stimuli, including trocar placement, diaphragmatic stimulation, and abdominal insufflation, can serve as potential triggers for AD, particularly when neuromuscular blockade is avoided to preserve diaphragmatic function, as was the case in this procedure [10]. Preventive measures were implemented, including Foley insertion to evacuate the bladder to minimize triggering stimuli, close hemodynamic monitoring with an arterial line, and readiness to treat hypertensive episodes with rapid-onset agents such as esmolol and nifedipine. While no episodes of AD occurred, proactive planning and monitoring for AD in high-level SCI patients undergoing procedures that involve visceral or somatic stimulation below the level of injury is imperative.

Arrhythmogenic complications are rare but notable. Dong et al. (2021) described a case of DP-induced ventricular tachycardia leading to cardiac arrest, which was resolved by adjusting pacemaker voltage. Although no arrhythmias occurred in our case, this report highlights the need for immediate readiness to address device-related complications [11]. Specifically, the interplay between cardiac pacemakers and diaphragmatic pacing requires careful consideration. Such interactions, although rare, raise concerns about potential misinterpretation of electrical signals when both cardiac and diaphragmatic devices are present. Preoperative interrogation of existing cardiac devices and intraoperative electrocardiographic monitoring are essential to mitigate these risks.

In summary, our case contributes to the limited but growing body of literature describing anesthetic management for DP placement in SCI patients. Our experience confirms several key themes from the literature: the importance of avoiding neuromuscular blockade, maintaining hemodynamic stability, continuous monitoring of diaphragmatic response, and anticipating potential complications such as DP-induced arrhythmia, AD, pneumothorax, diaphragmatic perforation, and phrenic nerve injury.

## Conclusions

As DP becomes an increasingly viable alternative to mechanical ventilation in patients with high cervical SCIs and other neuromuscular disorders, it is essential for anesthesiologists to understand the unique challenges and potential complications associated with this procedure. From preoperative optimization of respiratory mechanics to intraoperative management involving ventilator transitions, autonomic instability, and potential interference with cardiac pacemakers, each stage demands planning and vigilance to enhance patient outcomes. Notably, this report presents the first case of laparoscopic DP placement in our community hospital, highlighting both the evolving capabilities of regional centers and the importance of anesthetic preparedness in supporting advanced respiratory interventions.
